# Commentary: Parsing the Behavioral and Brain Mechanisms of Third-Party Punishment

**DOI:** 10.3389/fnins.2017.00374

**Published:** 2017-06-29

**Authors:** Anne-Marie Nußberger, Mary Montgomery, Yingyi Luo, Hongbo Yu

**Affiliations:** ^1^Department of Experimental Psychology, University of OxfordOxford, United Kingdom; ^2^Institute of Linguistics, Chinese Academy of Social SciencesBeijing, China; ^3^Faculty of Science and Engineering, Waseda UniversityTokyo, Japan; ^4^Center for Brain and Cognitive Sciences, Peking UniversityBeijing, China

**Keywords:** third-party punishment, fMRI neuroimaging, intention, consequences of actions, multivariate pattern analysis

Third-party punishment (TPP) is an important safeguard of social cooperation (Fehr and Gächter, 2002; Buckholtz and Marois, 2012). Examples range from a teacher sending their students out of classroom because they misbehaved, to a judge sending a person criminal to prison because they committed murder. Investigating the psychological and neural mechanisms of TPP decisions has significant practical implications, such as advising when and how third parties (e.g., judges) might be susceptible to affective and cognitive constraints inherent to human nature (Krueger and Hoffman, 2016).

Two types of information have been identified as crucial for TPP decisions, namely the *mental state* of the suspect and the consequential *harm* caused to the victim (Cushman, 2008; Schein and Gray, in press). Previous research has suggested that brain areas associated with mentalizing and affective processing are recruited during TPP, but determining the specific contribution of each of these areas has been a challenge. This is partly due to limitations of using scenario-based paradigms in fMRI (Buckholtz et al., 2008; Treadway et al., 2014). Furthermore, given the relatively low temporal resolution of fMRI (Serences, 2004), it has proven difficult to dissociate signals related to the respective processing of mental state and harm from signals related to their integration and translation into a specific punishment, as previous studies presented information related to these different aspects all at a time (an alternative solution to the problem of low temporal resolution is using electroencephalogram, see Yoder and Decety, 2014; Hesse et al., 2016). Moreover, prior designs have usually included only two levels of mental states (intentional vs. unintentional) and damage (harm vs. no harm), whereas both factors could vary greatly in real-world settings. Where different magnitudes of harm were actually included (e.g., Buckholtz et al., 2008; Krueger et al., 2014), formal analyses of the neural correlates for the varying levels of harm have been missing.

In a recent publication, Ginther et al. (2016) address these methodological challenges by introducing three key innovations to TPP research. First, their design effectively orthogonalizes three crucial components of TPP judgments,—namely harm and mental state processing, their integration, and the ultimate punishment decision—by presenting them at separate stages of a trial (Ginther et al., 2016, Figure 1). This is in contrast to previous scenario-based studies on TPP or moral judgment where these stages were not separated (Greene et al., 2004; Buckholtz et al., 2008; Young and Saxe, 2008). Additionally, the authors balanced the presentation order within each participant to dissociate each component's processing from their integration, which inevitably arises with sequential presentations of mental state and harm information.

Second, a parametric manipulation of mental state and harm allowed the authors to characterize response-profiles of brain areas sensitive to the respective components more precisely. For instance, activations in orbitofrontal cortex (OFC) and dorsolateral prefrontal cortex (DLPFC) were higher for harm than for mental state evaluations. However, activations in OFC were best accounted for by a quadratic relationship with level of harm suggesting that OFC activity reflects decision-difficulty (being greater for intermediate levels of harm). In contrast, activations in DLPFC were best accounted for by a linear response to level of harm (Ginther et al., 2016, Tables 3,4) which may reflect culpability as a different decision-aspect. Ginther et al.'s findings also indicate a possible need to reinterpret previous neuroimaging findings that have been solely based on main effect contrasts (e.g., Harm > No-Harm, Intentional > Unintentional, see Buckholtz et al., 2008; Young and Saxe, 2008; Treadway et al., 2014; Yu et al., 2015), as such contrast may miss out important and interesting response profiles.

Third, multivariate pattern analysis (MVPA) was used to dissociate spatially overlapping neural ensembles that serve different functions. In line with previous research (Buckholtz et al., 2008; Young and Saxe, 2008; Treadway et al., 2014), the authors found mental state and harm evaluations were associated with response in bilateral superior temporal sulcus (STS) and temporal parietal junction (TPJ)—core areas linked to representing others' cognitive and affective states (Van Overwalle, 2009). Crucially, by using MVPA, Ginther et al. could show that activation patterns in both TPJ and STS are differentially associated with mental state and harm evaluations (cf. Ginther et al., 2016, p. 9,428). This is an important advance over previous studies using univariate analyses that may not have the sensitivity to dissociate spatially overlapping but functionally distinct neural ensembles (Woo et al., 2014).

Moreover, MVPA proved useful in clarifying the function of DLPFC in punishment decisions. In line with prior studies that have suggested a consistent relationship between DLPFC and TPP (Knoch et al., 2006; Buckholtz et al., 2015), the authors found increased activation in right DLPFC during the decision stage. However, in contrast to previous findings (Buckholtz et al., 2008), activation strength in DLPFC did not correlate with level of punishment. The results from the MPVA offered a potential explanation for this, as they showed that level of punishment predicted *patterns* of DLPFC neural activity, rather than activation *strength* (Ginther et al., 2016; Figures 6B,C).

Moving forward, the design devised by Ginther et al. would also allow investigating how sequential presentation-orders of mental state and harm information may influence punishment decisions. It has been demonstrated that the way of presenting an action's consequences may influence the judgment of mental states underlying that action, a phenomenon named the “Knobe Effect” (Knobe, 2003). Thus, future studies could test how differences in narrative frames modulate punishment decision-formation. In regard to neuroimaging methodology and analysis, future studies could further broaden our understanding of the neural mechanisms in TPP by focusing more on the functional and effective brain networks involved in moral/legal judgment (e.g., Bellucci et al., 2017), using techniques such as psychophysiological interaction and Granger causality modeling. Another avenue future studies could pursue, is to combine the novel design presented by Ginther et al. with brain lesion or virtual lesion (e.g., transcranial magnetic stimulation) approaches to investigate the causal role of specific brain areas in TPP (e.g., Buckholtz et al., 2015; Glass et al., 2016). The separation of different processing stages could be further enhanced by adopting techniques that allow for higher temporal resolutions (e.g., electroencephalography or magnetoencephalography). Put in context with paradigms adopted in previous neuroimaging studies of TPP, a systematic meta-analysis comparing scenario-based (e.g., Buckholtz et al., 2008; Treadway et al., 2014; Ginther et al., 2016) and interaction-based (e.g., Feng et al., 2016) designs could shed light on the question in how far neurobiological processes are common across paradigms, or paradigm-specific and as such potentially not genuine signatures of TPP.

Overall, the work by Ginther et al. expands the scope of neuroscientific research on TPP by (i) insightfully revising the scenario-based paradigm such that different processing stages can be separated and by (ii) introducing sophisticated data analysis techniques to better characterize encoding profiles of relevant brain areas. The novel findings from this study provide empirical evidence for numerous theoretical accounts of the neural basis of TPP and raise intriguing and testable questions for future research: How do people move from integrated mental state and harm information to a definitive punishment choice? How do the affective components of harm evaluation, as well as our pre-existing social norms, bias the neural encoding of mental states (e.g., Knobe, 2003)? And what kind of scenario narration (in verbal or non-verbal form) could minimize such biases in the legal system? We believe that seeking answers to these questions will not only advance our understanding of the neurobiological basis of TPP, but also of other psychological faculties that both enable, and are cultivated by, the concept of justice (Rawls, 1971).

## Author contributions

AN, MM, YL, and HY have written the manuscript, AN and HY have revised the mansucript.

### Conflict of interest statement

The authors declare that the research was conducted in the absence of any commercial or financial relationships that could be construed as a potential conflict of interest.
